# Predictive value of elevated neutrophil-lymphocyte ratio for left ventricular systolic dysfunction in patients with non ST-elevated acute coronary syndrome

**DOI:** 10.12669/pjms.311.5967

**Published:** 2015

**Authors:** Adem Bekler, Gokhan Erbag, Hacer Sen, Emine Gazi, Sedat Ozcan

**Affiliations:** 1 Adem Bekler, MD, Department of Cardiology, Canakkale Onsekiz Mart University, School of Medicine, Canakkale/Turkey.; 2 Gokhan Erbag, MD, Department of Internal Medicine, Canakkale Onsekiz Mart University, School of Medicine, Canakkale/Turkey.; 3 Hacer Sen, MD, Department of Internal Medicine, Canakkale Onsekiz Mart University, School of Medicine, Canakkale/Turkey.; 4 Emine Gazi, MD, Department of Cardiology, Canakkale Onsekiz Mart University, School of Medicine, Canakkale/Turkey.; 5 Sedat Ozcan, MD, Department of Cardiovascular Surgery, Canakkale Onsekiz Mart University, School of Medicine, Canakkale/Turkey.

**Keywords:** Acute coronary syndrome, Ejection fraction, Myocardial infarction, Neutrophil-lymphocyte ratio, Systolic dysfunction

## Abstract

**Objective:**

We aimed to study the predictive value of the neutrophil-lymphocyte ratio (NLR) for left ventricular systolic dysfunction (LVSD) in patients with non ST-elevated acute coronary syndrome (NST-ACS).

**Methods:**

A total of 405 patients (mean age 62 years and 75% male) with NST-ACS were included in the study. The study population was divided into tertiles based on admission NLR values. The low, medium and high tertiles defined as NLR≤1.81 (n=135), 1.81<NLR≤3.2 (n=135) and NLR>3.2 (n=135), respectively.

**Results:**

The patients in the high NLR group were older (p<0.001), have higher rate of diabetes mellitus (p=0.028) and non-ST elevated myocardial infarction (NSTEMI) (p<0.001) and have lower left ventricular ejection fraction (LVEF) (p<0.001). Baseline WBC (p=0.02) and neutrophil (p<0.001) levels and NLR (p<0.001) were significantly higher, baseline hemoglobin (p=0.044), hematocrit (p=0.019) and lymphocyte (p<0.001) levels were significantly lower in the high NLR group. NLR was negatively correlated with LVEF in correlation analysis. An NLR >3.2 and age ≥70 were found to be an independent predictor of systolic dysfunction in multivariate analyses.

**Conclusion:**

An NLR >3.2 is a useful predictor for LVSD in patients with NST-ACS. The practice of using an NLR count on admission may be useful for identifying high-risk patients and their associated treatment methods.

## INTRODUCTION

Inflammation and inflammatory factors play a substantial role in the formation and progression of atherosclerotic plaque, and can lead to determinatively acute thrombotic complications of atheroma.^1^ Elevated leucocyte count is a marker for cardiovascular risk prediction, and the correlation between leucocyte count and the risk of cardiovascular disease has been recently demonstrated.^2^^,^^3^ However, although an increased leucocyte count is related to cardiovascular events and mortality in acute coronary syndromes (ACS), recent studies suggest that the neutrophil/lymphocyte ratio (NLR) is a more specific determiner than the neutrophil or leucocyte count.^4^

Left ventricular systolic dysfunction (LVSD) is an important cause both of mortality and morbidity in patients with ACS. In patients with ischemic or non-ischemic LVSD, the neutrophil count was demonstrated to be related with cardiovascular mortality.^5^ Recent studies showed that heart failure (HF) develops more frequently in patients with ST-elevated ACS who have a high NLR on admission to the hospital, and that HF can occur after either a short or long period after ACS.^6^ Here, we aimed to investigate the predictive value of NLR for LVSD in patients with NST-ACS.

## METHODS

Enrollments of patients with ACS described as unstable angina (UA) and non-ST elevated myocardial infarction (NSTEMI) who were admitted to our institution between March 2011 and August 2013 were evaluated retrospectively. Patients with clinical evidence of cancer, chronic inflammatory disease (CID), or any systemic infection were excluded. Therefore, 405 patients who were diagnosed with NST-ACS were enrolled. The patients were divided into tertiles based on admission NLR values. The low, medium and high tertiles defined as NLR≤1.81 (n=135), 1.81<NLR≤3.2 (n=135) and NLR>3.2 (n=135), respectively.

Diagnosis of UA and NSTEMI were defined according to American College of Cardiology guideline.^7^ Clinical characteristics and risk factors, i.e., smoking, hypertension (HT), and diabetes mellitus (DM) were obtained from the patients’ medical records. Patients treated with antihypertensive drugs or whose baseline blood pressure was over 140/90 mmHg were diagnosed with HT. Patients with DM were identified as pre-diagnosed and/or being on antidiabetic medication or newly diagnosed patients whose fasting plasma glucose level was ≥126 mg/dL or blood glucose level at any time was ≥200 mg/dL. LVSD was defined as ejection fraction ≤40% measured by transthoracic echocardiography on first admission to coronary care unit.

Total leukocyte count and its subtypes and biochemical values were appreciated retrospectively from blood samples and analyzed using a Beckman Coulter LH 780 (Beckman Coulter Ireland Inc. Mervue, Galway, Ireland) device in the laboratory of our institution.

Echocardiography was performed using a machine (Vivid 7®, GE Vingmed Ultrasound A/S, Horten, Norway) with a 3.5-MHz transducer. Left ventricular ejection fraction (LVEF) was assessed by Simpson’s method.

Angiographic data of the patients were evaluated from catheter laboratory records. All patients underwent a coronary angiography by the femoral approach using the standard Judkin’s technique. Iopromide as a contrast agent (Ultravist-370, Bayer Schering Pharma, Germany) and 6F diagnostic catheter were used in all patients.

All statistical analyses were performed using the SPSS program (version 17.0, SPSS, Chicago, IL, USA). Kruskal–Wallis variance analysis was used for comparisons of continuous variables. If there was a significance, Mann–Whitney U test was used for post hoc analysis. Quantitative variables were expressed as the median (interquartile range), and qualitative variables were expressed as percentages (%). Categorical variables were compared by chi-square test or Fisher exact test. A backward stepwise multivariate logistic regression analysis that included variables with p <0.1 was performed to identify independent predictors of LVSD. Age ≥70, NSTEMI, and NLR>3.2 were entered into the model. A p value <0.05 was accepted statistically significant.

## RESULTS

A total of 405 patients (304 males and 101 females) with NST-ACS (253 NSTEMI and 152 UA) were included in the study. The patients in the high NLR group were older (p<0.001), had a higher rate of DM (p=0.028) and NSTEMI (p<0.001) and a lower LVEF (p<0.001) than did the patients in the low and medium NLR groups. Table-I summarises the baseline characteristics of the groups.

Baseline leucocyte count (p=0.02), neutrophil count (p<0.001) and NLR (p<0.001) were significantly higher and baseline haemoglobin (p=0.044), hematocrit (p=0.019) and lymphocytes (p<0.001) levels were significantly lower in the high NLR group. Table-II shows the baseline laboratory findings of the groups.

**Fig.1:**
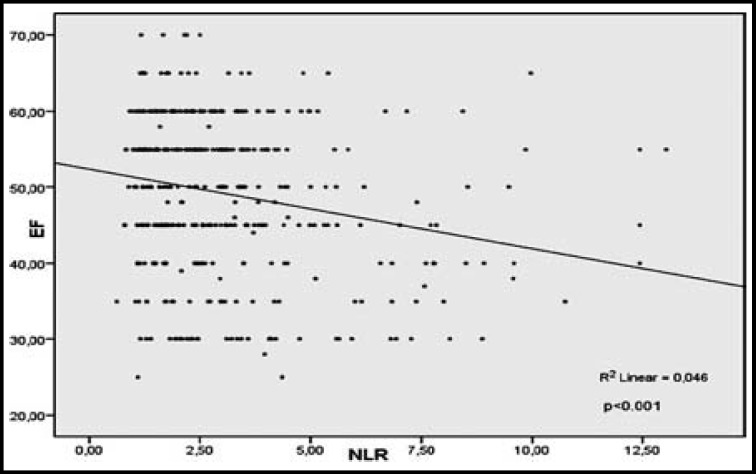
Relationship between left ventricular ejection fraction (EF) and neutrophil-lymphocyte ratio (NLR) in patients with non-ST elevated acute coronary syndrome (NST-ACS).

A correlation analysis found that NLR was negatively correlated with LVEF (r=−0.214, p<0.001). The relationship between LVEF and NLR in patients with NST-ACS is demonstrated in Fig.1.

Independent predictors of systolic dysfunction were determined using a backward stepwise multivariate logistic regression. Age ≥70 years, NSTEMI and NLR were found to be associated with systolic dysfunction. NLR>3.2 and age ≥70 years were found to be independent predictors of systolic dysfunction in multivariate analyses (HR:2.01, 95% CI:1.25–3.24, p=0.004; HR:1.90,95% CI:1.17–3.1, p=0.009, respectively). Table-III shows the results of univariate and multivariate analysis for risk factors of LVSD.

## DISCUSSION

The present study shows that an NLR >3.2 was associated with LVEF ≤40 on admission of patients with NST-ACS. The study focuses on patients with LVSD and ACS, because it has long been known that ACS is associated not only with mortality but also with morbidity, and, in particular, with congestive heart failure (CHF). When HF is related to coronary artery disease (CAD), patients have higher rate of mortality. Leucocytosis is one of the common findings in acute myocardial infarction (AMI), and differential analysis of leucocytes may give additional data for the determination of high risk patients, which is an important issue in daily practice.

The total leucocyte count can be rapidly evaluated within the first hour at an emergency department; thus, it is a useful marker for diagnosis and prognosis.^8^ Recent studies have demonstrated the predictive value of the leucocyte count in patients with AMI.^5^^,^^6^ Moreover, recent data have shown that certain specific leucocyte subtypes possess a greater predictive importance in the evaluation of all cardiovascular risk. Myocardial injury is followed by neutrophilia in ACS. It is also known that neutrophils can cause the release of certain substances such as proteolytic enzymes and superoxide radicals, and may thus play a role in atherosclerotic plaque rupture and aggravate the inflammatory condition. The association of neutrophils with tissue damage in MI patients has been postulated in some studies.^9^ Leucocyte-platelet aggregates may lead to vascular occlusion and be liable for infarct expansion in patients with ACS.^10^ Furthermore, microvascular obstruction with neutrophil-platelet plugs and vasoconstriction may cause the most extensive myocardial damage. Avanzas et al. demonstrated that a high neutrophil count is related to the extent of the infarct area in MI.^11^

However, a high NLR is an indicator of a high inflammatory process and is an independent predictor for LVSD in CAD. Avci et al. have documented the neutrophi lymphocytes ratio is related to the severity of idiopathic dilated cardiomyopathy.^12^ Lazaro et al. indicated that inflammatory markers are related to the functional class and prognosis in stable HF patients, and concluded that inflammation plays a key role in HF.^13^ Recent studies have shown advanced HF after MI and mortality were higher in patients with a higher neutrophil count.^14^

A higher NLR is associated with the severity of CAD^15^, and relative neutrophilia (together with lymphopenia) is associated with increased cardiovascular risk.^4^ Lymphopenia in relation to lymphocyte apoptosis indicates the presence of a highly inflammatory process^16^, and lymphopenia occurs in acute conditions due to lymphocyte apoptosis, thereby releasing pro-inflammatory cytokines due to the apoptotic cells.^17^ Dogdu et al. recently explained that a high NLR has a significant negative correlation with LVEF, and is an independent predictor of LVSD in stable multi-vessel CAD.^18^ Similarly, in our study, high NLR (>3.2) was an independent predictor of LVSD in patients with NST-ACS. Additionally, Sulaimen et al demonstrated that admission NLR is an independent predictor of all cause mortality in ACS patients.^19^ Our results suggest that a high NLR may relate to poor adaptive mechanisms in myocardial tissue, rather than to a culprit lesion or to multi-vessel disease. The presence of both neutrophilia and lymphopenia in patients indicate the presence of a highly inflammatory process, and inflammation is an important factor for myocardial damage in these patients.

In our study, patients with a high NLR were older and had a higher inflammatory burden due to co-morbities such as DM. To the best of our knowledge, the relationship between age and NLR has not yet been investigated, and no study has yet analysed the relationship between NLR and systolic dysfunction in patients with NST-ACS. We speculate that a more intensive treatment method using an invasive approach may be useful to reduce HF and mortality in patients with NST-ACS and a high NLR.

**Table-I:** The baseline characteristics of patients

**Variable**	**Low (NLR ≤ 1.81)** **( n = 135)**	**Tertiles of NLR** **Medium (1.81 < NLR ≤ 3.2)** **( n = 135)**	**High (NLR > 3.2)** **( n = 135)**	**p value**
Male % (n)	74.1 (100)	75.6 (102)	76.6 (102)	0.949
Age (yrs)	60 (30-82)	60 (36-88)	66 (19-90)	< 0.001
Previous use of ASA % (n)	51.1 (69)	45.9 (62)	41.5 (56)	0.283
Hypertension % (n)	44.4 (60)	45.2 (61)	53.3 (72)	0.268
Diabetes mellitus % (n)	28.9 (39)	22.2 (30)	37 (50)	0.028
Current smoker % (n)	37 (50)	39.3 (53)	33.3 (45)	0.593
BMI (kg/m2)	25.9 (18.3-42.5)	26.5 (15.9-38.5)	26.5 (18.3-37.9)	0.453
Previous CABG % (n)	12.6 (17)	10.4 (14)	7.4 (10)	0.415
Previous PCI % (n)	30.4 (41)	32.6 (44)	26.7 (36)	0.561
NSTEMI % (n)	48.9 (66)	58.5 (79)	80 (108)	< 0.001
Culprit lesion % (n)				0.253
LAD	38.5 (52)	40 (54)	42.2 (57)	
Cx	43 (58)	32.6 (44)	32.6 (44)	
RCA	18.5 (25)	27.4 (37)	25.2 (34)	
LV EF (%)	55 (25-70)	55 (30-70)	45 (25-65)	< 0.001

***Abbreviations:*** ASA, asetil salicylic acid; BMI, body mass index; CABG, coronary artery bypass grefting; Cx, circumflex; LAD, left anterior descending; LVEF, left ventricular ejection fraction;

NSTEMI, non ST elevation myocardial infarction; PCI, percutaneus coronary intervention;

RCA, right coronary artery.

**Table-II:** Patient's Laboratory Findings

**Variable**	**Low (NLR ≤ 1.81)** **( n = 135)**	**Tertiles of NLR** **Medium (1.81 < NLR ≤ 3.2)** **( n = 135)**	**High (NLR > 3.2)** **( n = 135)**	**p value**
T cholesterol (mg/dl)	195 (96-613)	195.4 (93.8-370)	192.2 (99.6-429.8)	0.693
LDL (mg/dl)	120 (39-220)	125 (11-312)	126 (41-286)	0.225
HDL (mg/dl)	39 (4-98)	40 (3-144)	41 (21-95)	0.653
Triglyceride (mg/dl) WBC (10^3^/mm^3^)	120 (40-1950) 9.4 (4.7-22.3)	120 (35-1024) 9.2 (3.8-16.4)	111 (32-649) 10.1 (4.8-19)	0.105 0.02
Hemoglobin (g/dL)	13.5 (8.1-16.7)	13.4 (8.1-17.1)	13 (7.7-17.4)	0.044
Hematocrit (%)	40.8 (25.5-50.3)	40.7 (24.8-52)	38.9 (22.1-53.3)	0.019
Platelet (10^3^/mm^3^)	222 (79-396)	241 (96-501)	231 (61-607)	0.168
RDW (%)	13.9 (12.3-19.6)	13.8 (12.1-23.6)	14.1 (11.7-18.7)	0.337
Neutrophil (10^3^/mm^3^)	4.9 (2.4-18.1)	5.86 (1.3-15.25)	7.91 (3.27-18)	< 0.001
Lymphocyte (10^3^/mm^3^)	3.56 (0.89-18)	2.4 (0.58-5.58)	1.58 (0.54-5)	< 0.001
NLR	1.38 (0.63-1.81)	2.43 (1.82-3.19)	4.4 (3.21-13.03)	< 0.001
MCV(fL)	88.8 (64-102.3)	89.5 (62.6-104.4)	88.7 (66.1-109.2)	0.698

***Abbreviations:*** HDL, high-density lipoprotein; LDL, low-density lipoprotein; MPV, mean platelet volume; MCV, mean corpusculer volume; NLR, neutrophil-lymhocyte ratio; PLT, platelet,

RDW, red cell distribution width; T, total; WBC, white blood cell.

**Table-III:** Univariate and Multivariate analysis for risk factors of left ventricular systolic dysfunction

	**Univariate**			**Multivariate** *		
**Variable**	**HR**	**(95% CI)**	**p**	**HR**	**(95% CI)**	**p**
DM	0.81	(0.50-1.32)	0.402			
Male	0.98	(0.58-1.66)	0.960			
Age ≥ 70 years	2.17	(1.35-3.49)	0.001	1.90	(1.17-3.1)	0.009
NSTEMI	1.79	(1.09-2.93)	0.020			
NLR > 3.2	2.25	(1.42-3.58)	0.001	2.01	(1.25-3.24)	0.004

***Abbreviations:*** CI, confidence interval; DM, diabetes mellitus; HR, hazard ratio;

NSTEMI, non ST elevated myocardial infarction; NLR, neutrophil-lymphocyte ratio;

*, analysis of Backward-Stepwise regression.

The present study has some limitations. First, our study was performed retrospectively and based on a relatively small group of patients. Second, one of the most important limitations was the failure to measure the LVEF of patients before their admission to hospital. Third, clopidogrel may cause neutropenia, and results may be altered in patients who have previously taken clopidogrel; however, our data were insufficient to identify clopidogrel use. Finally, we did not evaluate inflammation parameters such as high sensitivity CRP and MMP-9 that could be helpful in evaluating a high NLR.

## CONCLUSION

A high NLR (>3.2) is useful predictor for LVSD in patients with NST-ACS. Total and differential leucocyte count analyses are basic and inexpensive methods for use in evaluating patients with ACS; therefore, the practice of using an NLR count on admission may be useful for identifying high-risk patients and their associated treatment methods.

## Author’s Contributions:

**AB, GE and EC:** Conceived, designed, did statistical analysis and editing of manuscript.

**GE, HS and SO:** Did data collection and manuscript writing.

**AB, EG and SO:** Did review and final approval of manuscript.

**AB:** Takes the responsibility and is accountable for all aspects of the work in ensuring that questions related to the accuracy or integrity of any part of the work are appropriately investigated and resolved.

## Notes

***Source of funding:*** None.
